# Predictors of cough resolution following endoscopic minimally invasive treatment in patients with GERD and chronic cough: a retrospective study

**DOI:** 10.3389/fmed.2026.1796830

**Published:** 2026-05-29

**Authors:** Xinhui Fang, Lida Zhang, Songze Ding

**Affiliations:** Department of Gastroenterology and Hepatology, Henan Provincial People’s Hospital, Zhengzhou, Henan, China

**Keywords:** chronic cough, cough resolution, gastroesophageal reflux disease, minimally invasive, predictive factors

## Abstract

**Objectives:**

To investigate factors influencing cough resolution following endoscopic minimally invasive treatment in patients with gastroesophageal reflux disease (GERD) complicated by chronic cough, and to establish a predictive model based on these findings.

**Methods:**

Clinical data from 200 patients with GERD and cough undergoing endoscopic minimally invasive treatment at our hospital between January and December 2024 were included. All patient data were extracted from the electronic medical record system. Patients were divided into a cough non-relief group (*n* = 35) and a cough-relief group (*n* = 165) based on validated cough score reduction criteria (≥50% reduction in total cough score) post-treatment. Clinical data were collected and compared. Two machine learning methods-Least Absolute Subtraction and Selection (LASSO) regression and Extreme Gradient Boosting (XGBoost)-were employed for “intersection screening” of risk factors. Multivariate logistic regression analyzed risk factors for persistent cough after endoscopic minimally invasive treatment in GERD patients with chronic cough. A regression model was established using R software, validated internally via Bootstrap resampling (1,000 iterations), and evaluated for discriminatory power and consistency using receiver operating characteristic (ROC) curve area under the curve (AUC) and calibration curves.

**Results:**

Compared to the cough-relief group, the non-relief group exhibited significantly higher age, smoking history, cough duration, ineffective esophageal motility, DeMeester score, esophageal dysfunction, pepsin, and exhaled nitric oxide (FeNO), along with lower secretory immunoglobulin A (SIgA) levels (*P* < 0.05). LASSO regression and XGBoost with “overlapping coverage” identified seven common risk factors: age, cough duration, ineffective esophageal motility, DeMeester score, pepsin, FeNO, and SIgA. Multivariate logistic regression revealed that ineffective esophageal motility, cough duration, DeMeester score, pepsin, and FeNO were risk factors for persistent cough after endoscopic minimally invasive therapy (OR = 27.090, 2.639, 1.193, 1.106, 1.295, *P* < 0.05), while SIgA was a protective factor (OR = 0.891, *P* < 0.05). The Hosmer-Lemeshow test yielded χ^2^ = 1.535, *P* = 0.992 > 0.05, indicating good model calibration. A nomogram model was constructed based on multivariate regression analysis results. Model validation was performed using Bootstrap sampling (1,000 iterations), yielding a ROC-AUC of 0.967 (95% CI 0.936–0.998). The calibration curve demonstrated good agreement between predicted and actual values.

**Conclusion:**

Independent risk factors influencing persistent cough after endoscopic minimally invasive treatment in GERD patients with chronic cough include ineffective esophageal motility, cough duration, DeMeester score, SIgA, pepsin, and FeNO. The nomogram model constructed based on these factors demonstrates good predictive efficacy for clinical treatment outcomes.

## 1 Introduction

Gastroesophageal reflux disease (GERD) involves the reflux of gastric and duodenal contents into the esophagus. It is a common digestive disorder characterized by lower esophageal sphincter (LES) dysfunction and reduced esophageal clearance capacity (1, 2). Ahmed Z et al. (3) noted that chronic cough is one of the most common reasons for respiratory specialty clinic visits. GERD has been identified as a key factor causing chronic cough, with up to 20%–40% of chronic cough cases attributable to GERD, termed gastroesophageal reflux cough (GERC). However, the clinical diagnosis of GERC is particularly difficult because over 75% of patients do not present with typical digestive symptoms such as acid reflux or heartburn. Instead, they primarily exhibit respiratory symptoms like coughing, throat discomfort, or asthma-like symptoms (4). Conventional respiratory medicine approaches to suppressing or treating coughs often yield poor results (5).

Currently, endoscopic minimally invasive therapies have become mainstream for GERD (6). Ikoma et al. (7) propose that endoscopic minimally invasive procedures aim to physically enhance the barrier function of the LES and reduce transient relaxation, thereby anatomically and functionally correcting the root cause of reflux. However, endoscopic minimally invasive therapy imposes stringent patient selection criteria and faces inherent technical limitations, resulting in significant variability in postoperative cough relief among patients (8, 9). Therefore, accurately identifying high-risk groups and implementing corresponding interventions is crucial for enhancing treatment success rates. However, few studies have reported on predictors of cough relief following endoscopic minimally invasive treatment in patients with GERD and chronic cough.

This study aimed to identify predictors of cough resolution after endoscopic minimally invasive treatment in patients with GERD and chronic cough, and to establish a predictive model to support preoperative patient screening and personalized treatment decision-making.

## 2 Materials and methods

### 2.1 Ethical statement

This study was approved by the institutional review board and ethics committee. Given its retrospective nature and use of de-identified patient data, informed consent was not required as no risk or detriment to patients was anticipated. This exemption complies with regulations and ethical guidelines pertaining to retrospective research.

### 2.2 Study design

This retrospective study included clinical data from 200 patients with GERD and cough who underwent endoscopic minimally invasive treatment at our hospital between January and December 2024. The diagnosis of GERD was established according to the modern diagnostic criteria outlined in the Lyon Consensus 2.0 (2023) (10). This consensus defines conclusive evidence for GERD as meeting one of the following criteria in patients with typical or relevant symptoms: (1) endoscopic findings of Los Angeles (LA) grade B or above reflux esophagitis, biopsy-confirmed Barrett’s esophagus, or peptic esophageal stricture; or (2) an esophageal distal acid exposure time (AET) of >6% on 24-h pH or pH-impedance monitoring performed off antisecretory therapy. All patient data were extracted from the electronic medical record system. Patients were categorized into a cough non-relief group (*n* = 35) and a cough-relief group (*n* = 165) based on cough resolution following treatment.

### 2.3 Inclusion and exclusion criteria

Inclusion criteria: (1) Patients who meet the diagnostic criteria for GERD; (2) Met criteria for chronic cough and pulmonary manifestations in laryngopharyngeal reflux disease, characterized by predominantly daytime coughing with occasional nocturnal coughing (11); (3) Age > 18 years; (4) All patients underwent either fundoplication or radiofrequency ablation (Stretta) of the LES according to disease severity, not both procedures uniformly; (5) Eligibility for endoscopic minimally invasive treatment; (6) Complete relevant clinical documentation.

Exclusion criteria: (1) Severe renal insufficiency; (2) History of neurological disorders; (3) History of gastrointestinal surgery; (4) Concurrent malignant tumors; (5) Cough caused by severe cardiopulmonary disease, upper respiratory tract infection, asthma, chronic bronchitis, etc.; (6) Women who are lactating, pregnant, or planning pregnancy in the near future.

### 2.4 Endoscopic minimally invasive treatment methods

All procedures were performed by three senior gastroenterologists with more than 10 years of experience in endoscopic anti-reflux procedures, using a standard electronic gastroscope (GIF-H260, Olympus, Japan) under intravenous sedation with propofol.

(1) Preoperative preparation: Routine laboratory tests including blood routine, coagulation function, liver and kidney function, and electrocardiography were completed to exclude contraindications. All patients underwent high-definition gastroscopy to evaluate the grade of reflux esophagitis, hiatal hernia size, and cardiac relaxation. Written informed consent was obtained from all patients before the procedure, detailing the purpose, process, benefits, and potential risks.

(2) Surgical procedures: Patients were placed in the left lateral decubitus position. According to the preoperative evaluation of GERD severity, esophageal motility, and hiatal hernia, one of the following two standardized procedures was performed: (1) Radiofrequency ablation (stretta procedure): For patients with LA grade B GERD, no significant hiatal hernia (<2 cm), and preserved esophageal peristalsis, radiofrequency ablation of the lower esophageal sphincter (LES) was performed. A Stretta catheter was inserted transorally into the esophagus, with the electrode positioned 0.5–1 cm above the gastroesophageal junction. Radiofrequency energy (60 °C–65 °C, 10–15 W) was delivered circumferentially at four levels to induce local thermal injury and submucosal fibrosis, thereby increasing LES pressure, reducing transient LES relaxation, and improving anti-reflux barrier function. (2) Endoscopic cardiac sphincter reformation (PECC): For GERD Grade C–D patients, or Grade B patients with hiatal hernias > 2 cm, endoscopic esophageal sphincter reduction (PECC) is performed. Depending on the degree of sphincter relaxation, 2–5 endoscopic ligation rings are applied around the upper and lower edges of the cardia. The number and depth of ligation were adjusted according to the degree of sphincter relaxation. If the anti-reflux effect was insufficient during intraoperative assessment, supplementary ligation or secondary reoperation after 4 weeks was arranged. (3) Postoperative management: Bed rest for 12 h postoperatively; avoid vigorous coughing for 24 h and strenuous physical activity for 1 week. Maintain fasting for 1 day, followed by a liquid diet for 3 days. Avoid spicy or irritating foods and alcohol. Patients with lower esophageal or cardia erosions may receive oral thrombin powder as indicated, along with proton pump inhibitors and mucosal protectants for 2 weeks. Antibiotics are generally not required. Patients on anticoagulants should resume medication no sooner than 5 days postoperatively. (4) Emergency plan and management measures: Monitor bleeding status; perform endoscopic hemostasis if necessary. Assess epigastric pain severity; mild pain may resolve spontaneously, but administer analgesics if required.

### 2.5 Scoring criteria

Assessment follows the Clinical Practice Guidelines: Chronic Cough (12) using a five-point scale. Daytime cough symptom scores are defined as: 0 points: No cough; 1 point: 1–2 brief cough episodes; 2 points: ≥2 brief cough episodes; 3 points: Frequent coughing without impacting daily activities; 4 points: Frequent coughing affecting daily activities; 5 points: Frequent coughing preventing normal daily activities. Nighttime cough symptom scoring: 0 points: No coughing; 1 point: Coughing only while awake or upon falling asleep; 2 points: Coughing causing 1 episode of awakening or early morning awakening; 3 points: Coughing causing multiple nighttime awakenings; 4 points: Coughing for most of the night; 5 points: Severe coughing preventing sleep. All patients underwent a 12-month follow-up post-treatment. The definition of cough relief is: During the follow-up period, the total cough score during both daytime and nighttime is reduced by ≥50% compared to the baseline level, and the cough frequency decreases and the number of attacks reduces; The definition of unrelieved cough is: The score reduction is less than 50% or the patient continues to have severe cough or frequent cough attacks. Objective 24-h esophageal pH-impedance monitoring was performed to confirm reflux reduction as the mechanism of symptom improvement.

### 2.6 Clinical data collection

Clinical data were collected based on systematic medical records, including gender (male/female), age, body mass index (BMI), educational attainment (junior high school or below/high school or above), smoking history (daily consumption > 1 cigarette, with duration > 1 year or cessation < 1 year) (yes/no), alcohol consumption history (daily intake > 1 drink unit, duration > 1 year or abstinence < 1 year; 1 drink unit = 45 mL baijiu/360 mL beer/120 mL fruit wine) (yes/no), Hypertension [meeting criteria in The Japanese Society of Hypertension Guidelines for Self-monitoring of Blood Pressure at Home (Second Edition) (13)] (Yes/No), Diabetes [meeting criteria in Application of the Chinese Expert Consensus on Diabetes Classification in Clinical Practice (14)] (Yes/No), History of reflux esophagitis (Yes/No), Total reflux episodes, Longest reflux duration, Cough duration, Presence of typical reflux symptoms (Yes/No), Cough threshold, Lower esophageal sphincter pressure (LESP), Upper esophageal sphincter pressure (UESP), Integrated relaxation pressure over 4 seconds (IRP4s), Distal contractile pattern (DCI), distal latency (DL), ineffective esophageal motility (yes/no), esophageal acid exposure composite score (DeMeester score), esophageal dysfunction (presence/absence).

### 2.7 Laboratory parameters

Collect patients’ morning fasting saliva samples as follows: Rinse mouth before collection, expel residual saliva, forcefully spit out pharyngeal saliva into a 0.5 mL test tube containing 0.1 mol/L citrate. Collected saliva was stored at 4 °C. Centrifuge at 12,000 rpm to obtain supernatant, which was stored for subsequent testing. Salivary secretory immunoglobulin A (SIgA) and pepsin were measured using enzyme-linked immunosorbent assay (ELISA) kits from Shanghai Enzyme-Linked Bio-Research Institute, strictly following the manufacturer’s instructions. Exhaled nitric oxide (FeNO) was measured using a NO analyzer.

### 2.8 Statistical analysis

Data were analyzed and visualized using SPSS 26.0 and STATA 17.0. Normality of distribution for quantitative data was assessed using the Shapiro-Wilk test. Normally distributed quantitative data underwent independent samples *t*-tests and are presented as ($\bar{X}$ ± s). Non-normally distributed data underwent Mann-Whitney U tests, expressed as [M (P25, P75)] and subjected to Z-tests; categorical variables underwent chi-square (χ^2^) tests and were presented as frequencies, with a significance level of *P* < 0.05. LASSO regression was used for linear feature selection with 10-fold cross-validation to avoid multicollinearity; XGBoost was applied with adjusted parameters (learning rate = 0.1, max depth = 3, subsample = 0.8) to reduce overfitting risk in small samples; feature importance threshold was determined based on 10-fold cross-validation results rather than a fixed 5% cutoff. Selected factors were incorporated into logistic regression analysis. A regression prediction model was constructed, and its discriminatory power and calibration were evaluated using the area under the receiver operating characteristic (ROC) curve (AUC) and calibration curves.

## 3 Results

### 3.1 Comparison of clinical data and laboratory indicators between the non-cough relief group and the cough relief group

Comparisons of gender, body mass index, educational level, and other characteristics between the cough non-relief group and the cough-relief group showed no statistically significant differences (*P* > 0.05). Compared with the cough-relief group, the cough non-relief group had higher mean age, smoking history, cough duration, ineffective esophageal motility, DeMeester scores, esophageal dysfunction, pepsin, and FeNO levels, while SIgA levels were lower (*P* < 0.05), as shown in Table 1.

**TABLE 1:** Comparison of clinical data and laboratory indicators between the non-cough relief group and the cough relief group.

Indicator		Cough non-relief group (*n* = 35)	Cough relief group (*n* = 165)	*t/x* ^2^	*P*
Gender (*n*)	Male	20	85	0.367	0.545
	Female	15	80		
Age (years, $\bar{X}$ ± s)		67.78 ± 14.88	55.34 ± 13.21	4.947	<0.001
BMI (kg/m^2^, $\bar{X}$ ± s)		23.95 ± 2.41	23.81 ± 2.52	0.301	0.764
Educational attainment (*n*)	Junior high school and below	21	78	1.871	0.171
	High school and above	14	87		
History of smoking (*n*)	Have	18	43	8.766	0.003
	No	17	122		
History of alcohol consumption (*n*)	Have	12	33	3.379	0.066
	No	23	132		
Hypertension (*n*)	Have	15	45	3.340	0.068
	No	20	120		
Diabetes (*n*)	Have	9	29	1.243	0.265
	No	26	136		
History of reflux esophagitis (*n*)	Have	4	8	2.217	0.137
	No	31	157		
Total number of backflows (times, $\bar{X}$ ± s)		55.34 ± 6.71	53.89 ± 6.34	1.216	0.225
Maximum backflow time (min, $\bar{X}$ ± s)		32.12 ± 4.85	30.99 ± 3.32	1.673	0.096
The course of cough (month, $\bar{X}$ ± s)		6.34 ± 1.56	4.86 ± 1.39	5.598	<0.001
Accompanied by typical reflux symptoms (*n*)	Yes	25	111	0.229	0.631
	No	10	54		
Cough threshold (μmol/L, $\bar{X}$ ± s)		0.61 ± 0.22	0.57 ± 0.23	0.941	0.348
LESP (mmHg, $\bar{X}$ ± s)		18.95 ± 3.41	19.22 ± 3.56	0.410	0.682
UESP (mmHg, $\bar{X}$ ± s)		29.69 ± 4.12	30.18 ± 4.44	0.600	0.549
IRP4s (mmHg, $\bar{X}$ ± s)		7.91 ± 1.15	8.05 ± 1.22	0.623	0.534
DCI (mmHg⋅s⋅cm, $\bar{X}$ ± s)		564.77 ± 66.53	569.89 ± 53.83	0.489	0.625
DL (s, $\bar{X}$ ± s)		574.85 ± 59.61	559.69 ± 60.23	1.355	0.177
Ineffective esophageal motility (*n*)	Yes	14	27	9.898	0.002
	No	21	138		
DeMeester score (points, $\bar{X}$ ± s)		53.15 ± 6.93	45.69 ± 8.16	5.035	<0.001
Esophageal dysfunction (*n*)	Have	15	35	7.215	0.007
	No	20	130		
SIgA (mg⋅L^1^, $\bar{X}$ ± s)		61.37 ± 7.29	71.89 ± 9.47	6.190	<0.001
Pepsin (μg⋅L^1^, $\bar{X}$ ± s)		119.47 ± 13.84	103.84 ± 15.12	5.634	<0.001
FeNO (×10^9^, $\bar{X}$ ± s)		45.26 ± 5.05	39.39 ± 5.36	5.942	<0.001

### 3.2 LASSO and XGBoost screening of key variables influencing cough relief after treatment for GERD with chronic cough

**FIGURE 1:**
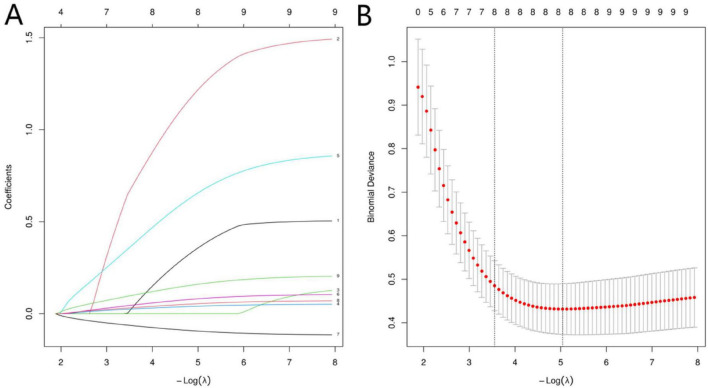
Least Absolute Subtraction and Selection (LASSO) regression analysis for feature selection in cough resolution prediction. **(A)** Coefficients. **(B)** Binomial deviarnce.

**FIGURE 2:**
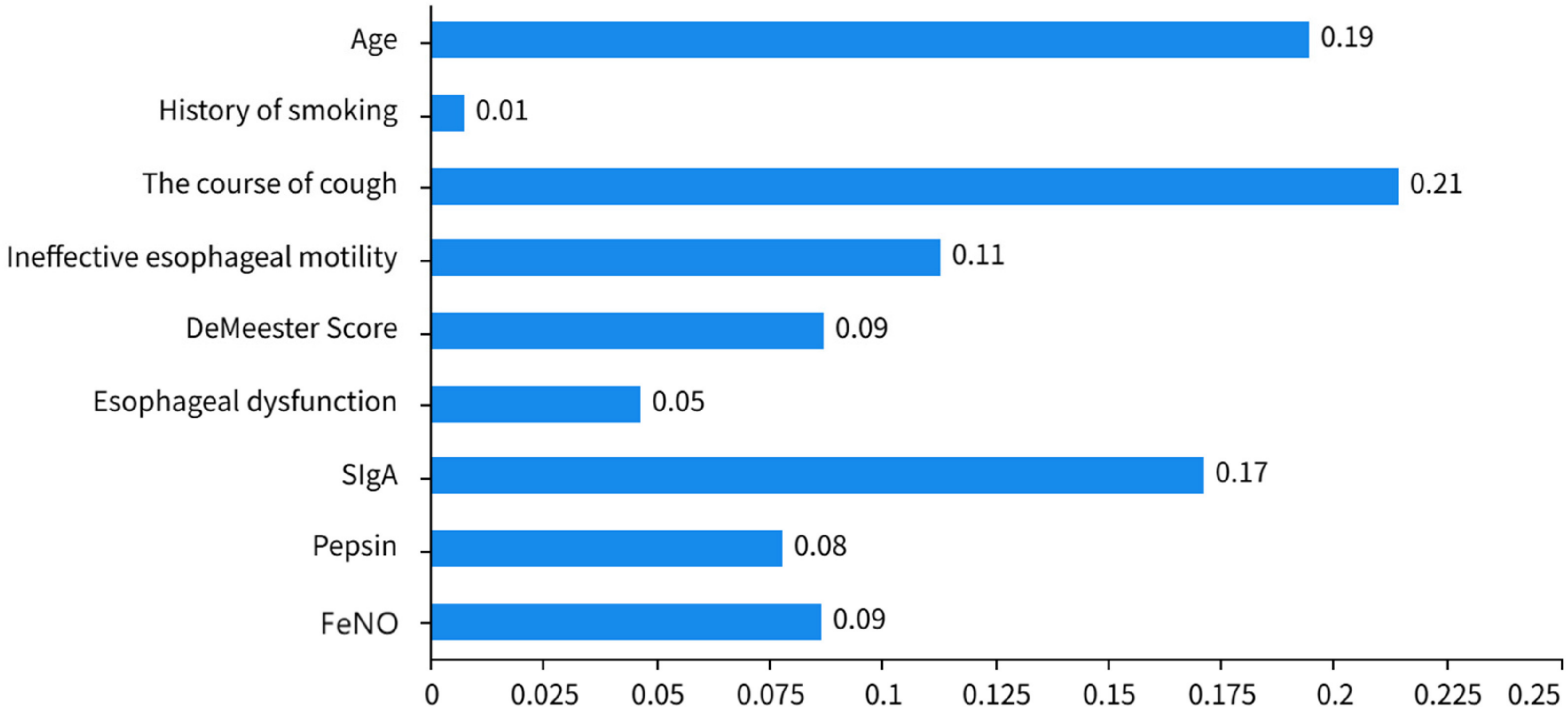
Extreme Gradient Boosting (XGBoost) model feature importance analysis for cough resolution prediction.

To optimize model performance and address multicollinearity, clinically significant variables identified by univariate analysis (*P* < 0.05) were incorporated into LASSO regression for feature selection. Regression coefficients of redundant variables were compressed to zero. The process for determining the optimal λ (K value) is as follows: The dataset of variables identified as significant in univariate analysis (containing all candidate features) was randomly divided into non-overlapping subsets. A total of 10-fold cross-validation was employed to evaluate model fit across different λ values, generating cross-validation plots [log(λ) on the x-axis, model mean squared error MSE on the y-axis]. Through K-fold cross-validation, the optimal LASSO regression penalty coefficient λ = 0.006 was determined, incorporating eight variables: age, smoking history, cough duration, ineffective esophageal motility, DeMeester score, pepsin, FeNO, and SIgA (Figure 1). XGBoost model parameters were set as follows: Learning rate: 0.1, maximum depth: 6, subsampling ratio: 0.8, number of generations: 200. Feature importance was assessed using the “gain” metric. Feature importance threshold was determined by 10-fold cross-validation optimization. First, the percentage of each feature’s gain value relative to the total sum of all features’ gains was calculated. To identify variables with significant predictive capability, referencing commonly used threshold ranges for feature importance screening in similar studies (typically 5%–15%) and considering the distribution of feature importance in this study, a cutoff value of <5% importance was set. Features with importance with low marginal contribution were excluded. Following XGBoost analysis, the smoking history importance was low and thus excluded. Ultimately, eight important variables were included: age, esophageal dysfunction, cough duration, ineffective esophageal motility, DeMeester score, pepsin, FeNO, and SIgA. The two machine learning models’ parallel evaluations of important variables underwent “overlap coverage” analysis, jointly identifying seven meaningful factors: age, cough duration, ineffective esophageal motility, DeMeester score, pepsin, FeNO, and SIgA (Figure 2).

### 3.3 Multivariate logistic regression analysis of persistent cough following endoscopic minimally invasive treatment in patients with GERD and chronic cough

Variables selected by LASSO and XGBoost machine learning were incorporated into logistic regression analysis. Results indicated that ineffective esophageal motility, cough duration, DeMeester score, pepsin, and FeNO were all risk factors for persistent cough after endoscopic minimally invasive treatment (OR = 27.090, 2.639, 1.193, 1.106, 1.295, *P* < 0.05), while SIgA was a protective factor (OR = 0.891, *P* < 0.05). The Hosmer-Lemeshow goodness-of-fit test indicated that the null hypothesis for this model was accepted, as the model fit value and observed value showed consistent agreement (χ^2^ = 1.535, *P* = 0.992 > 0.05). This confirms the model passed the H-L test with good calibration, as shown in Table 2.

**TABLE 2:** Multivariate logistic regression analysis of cough not relieved in patients with gastroesophageal reflux disease (GERD) complicated with chronic cough after minimally invasive endoscopic treatment.

Variables	β	S.E	Z	*P*	OR (95% CI)
Ineffective esophageal motility	3.299	1.017	3.244	0.001	27.090 (3.690∼198.862)
Age	0.049	0.033	1.483	0.138	1.051 (0.984∼1.121)
The course of cough	0.970	0.342	2.840	0.005	2.639 (1.351∼5.155)
DeMeester score	0.176	0.068	2.603	0.009	1.193 (1.045∼1.362)
SIgA	−0.116	0.044	−2.613	0.009	0.891 (0.817∼0.971)
Pepsin	0.101	0.037	2.712	0.007	1.106 (1.028∼1.190)
FeNO	0.259	0.084	3.076	0.002	1.295 (1.098∼1.527)

### 3.4 Development of a regression model for predicting persistent cough after endoscopic minimally invasive treatment in patients with GERD and chronic cough

**FIGURE 3:**
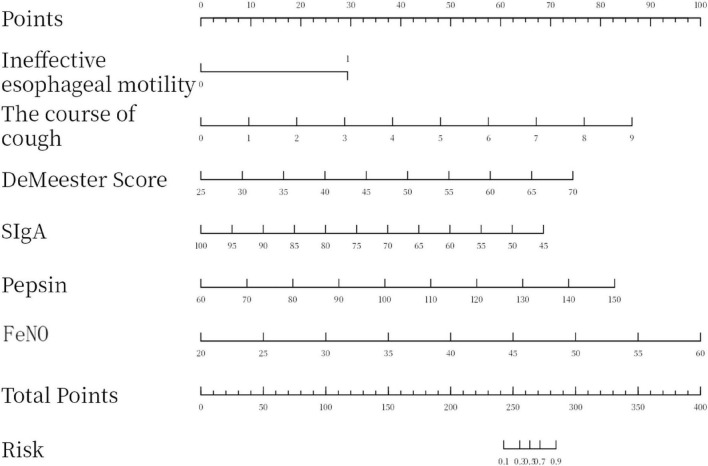
Nomogram model for predicting the risk of cough non-relief after endoscopic minimally invasive treatment.

Based on the aforementioned statistical analysis results, the following regression model was established using multivariate adjusted coefficients: In(p/1−p) = −24.549 + 3.299 × ineffective esophageal motility + 0.970 × cough duration + 0.176 × DeMeester score–0.116 × SIgA + 0.101 × pepsin + 0.259 × FeNO. Using the six independent predictors identified through screening, a visual scoring system was developed. Each risk factor corresponds to an independent scale axis, where the scale length intuitively reflects the factor’s contribution weight to the occurrence of persistent cough after endoscopic minimally invasive treatment in patients with GERD and chronic cough. The total score is calculated by summing the values corresponding to each variable, and the final risk probability is obtained by mapping this total score onto a probability scale, as shown in Figure 3.

### 3.5 Model performance evaluation

**FIGURE 4:**
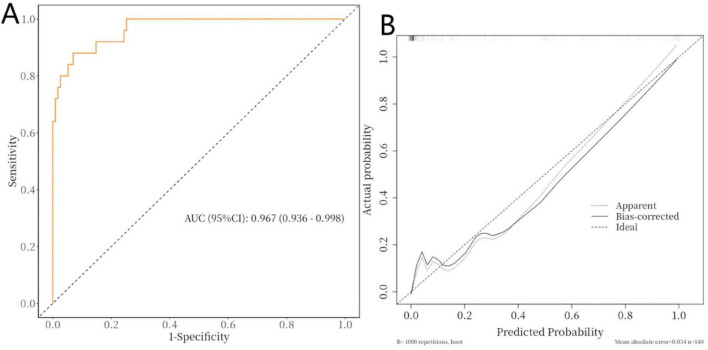
Receiver operating characteristic (ROC) curve **(A)** and calibration curve **(B)** for validation of the predictive model.

The model was validated using 1,000 bootstrap resamples. The ROC curve revealed an AUC value of 0.967 (95% CI 0.936–0.998), indicating that the modified line chart model exhibits excellent discriminatory capability (see Figure 4A). The calibration curve shows Cox-Snell R^2^ = 0.440 and Nagelkerke R^2^ = 0.728, indicating strong explanatory power for the dependent variable and good agreement between predicted and actual values (see Figure 4B). The high AUC is attributed to strict feature screening and homogeneous single-center population.

## 4 Discussion

Previous data indicate that patients with severe GERD complicated by chronic cough often undergo laparoscopic fundoplication and esophagogastric anastomosis, but these procedures carry high treatment costs and low patient acceptance. For mild to moderate cases, drug therapy yields poor efficacy, frequent complications, and suboptimal clinical outcomes (15, 16). Endoscopic minimally invasive therapy significantly reduces treatment complexity and costs while improving clinical efficacy for GERD patients with chronic cough. However, its effectiveness exhibits heterogeneity, and cough persistence remains in some patients due to multiple interacting factors, adversely affecting their health and quality of life. Therefore, actively analyzing factors associated with persistent cough after endoscopic minimally invasive treatment is crucial for early identification of high-risk groups, implementation of preventive measures, and improved outcomes.

The results of this study indicate that the relief of cough after endoscopic minimally invasive treatment is closely related to six factors: poor esophageal motility function, duration of cough, Demetris score, SIgA, pepsin, and FeNO. Among them, poor esophageal motility function is the most powerful predictor of poor cough relief. This is because poor esophageal motility function directly reduces the patient’s esophageal clearance ability, causing prolonged retention of reflux substances, continuous stimulation of the pharynx and airway mucosa, and maintaining the cough reflex. The endoscopic operation cannot completely reverse severe motility disorders, thus leading to persistent cough after the operation (17). Secondly, a longer duration of cough also affects the relief of cough. This is mainly attributed to the long-term cough of the patient causing chronic inflammation, fibrosis, and remodeling of the pharyngeal mucosa structure, resulting in irreversible and irreparable damage, thereby weakening the effect of cough relief (18). Additionally, a higher Demetris score indicates more severe gastric acid reflux and longer mucosal exposure time, which causes extensive and continuous damage to the esophagus and pharynx. Patients with higher scores often have more severe lower esophageal sphincter function disorders, which limit the effect of endoscopic anti-reflux surgery and lead to persistent cough (19). Furthermore, SIgA in saliva plays a protective role, effectively promoting the effect of cough relief. SIgA can maintain the integrity of the mucosal barrier, inhibit inflammatory responses, and reduce the sensitivity of the mucosa to reflux stimulation; a lower level of SIgA weakens the defense and repair capabilities of the mucosa, increasing the risk of persistent cough (20). Moreover, elevated pepsin levels are closely related to the poor prognosis of cough relief in patients. This is because pepsin reflects non-acidic reflux and micro-inhalation, directly corroding the mucosa, activating cough receptors, and enhancing airway hyperresponsiveness; the higher the pepsin level, the more severe the esophageal external injury, resulting in poor cough relief effect. Additionally, a higher FeNO level indicates the presence of persistent airway inflammation and hyperreactivity, and its increase is closely related to persistent cough after endoscopic treatment (21).

Analysis of this study’s data reveals that compared to the cough-relief group, the cough non-relief group exhibited higher levels of pepsin and FeNO, along with lower SIgA levels. These factors collectively constitute risk indicators for persistent cough following endoscopic minimally invasive treatment (*P* < 0.05). Previous studies indicate that pepsin is a crucial enzyme that breaks down proteins in food into small peptide molecules for human absorption. Normally present in gastric juice, detectable levels in saliva indicate the presence of gastric reflux (22, 23). Gu et al. (24) suggested that GERD with chronic cough primarily relates to airway microaspiration, esophagogastric-bronchial reflex, and esophageal dysmotility. Refluxate includes both acidic and non-acidic components. Persistent cough symptoms in GERD patients with chronic cough despite treatment indicate a strong association between cough and non-acidic reflux. Pepsin, a primary component of gastric acid reflux, poses significant respiratory hazards. As an acid-activated digestive enzyme, it exhibits strong mucosal corrosivity. Elevated pepsin levels entering the pharynx directly compromise the mucosal barrier, exacerbate airway hyperresponsiveness, activate cough receptors, and readily trigger the cough reflex, thereby increasing postoperative cough risk. Research indicates that NO possesses potent antiviral and immune-enhancing properties, playing a crucial role in regulating nasal respiration. Elevated exhaled NO increases blood flow velocity within the nasal cavity and excessively accelerates mucosal fiber activity, leading to vasodilation and abnormal glandular secretion (25). FeNO, a biomarker produced by airway epithelial cells, exhibits a strong correlation between its concentration and inflammatory cell counts. It reflects non-bacterial allergic inflammation levels and serves as a key indicator of airway inflammation, widely applied in clinical assessments of respiratory diseases (26). Elevated FeNO levels in GERD patients with chronic cough indicate concomitant chronic airway inflammation, immune dysregulation, weakened mucosal defense functions, delayed reflux clearance, and heightened airway hyperresponsiveness. This further stimulates cough receptors, increasing sensitivity to minimal reflux and elevating postoperative cough risk. Previous studies indicate that mucosal barriers constitute the body’s primary immune defense. Salivary immunoglobulin A (SIgA), the predominant antibody in saliva, specifically binds to viruses and bacteria, preventing their adhesion to mucosal surfaces and blocking infection pathways. Elevated SIgA levels also control inflammatory spread, protecting the respiratory tract from bacterial and viral invasion, thereby playing a crucial role in immune function (27). In patients with GERD and chronic cough, the irritation caused by gastroesophageal reflux contents leads to pharyngeal inflammation. Prolonged chronic inflammation compromises the body’s immunity. When the airway mucosal epithelium is damaged, its ability to secrete SIgA decreases, leading to reduced salivary SIgA levels. This weakens the mucosal barrier, delays reflux clearance, exacerbates airway hyperresponsiveness, and damages the pharynx, increasing the risk of unresolved postoperative cough in patients.

Traditional logistic regression is susceptible to multicollinearity and overfitting, leading to the loss of critical predictive information in models. This study innovatively combines machine learning with multivariate regression. On one hand, L1 regularization via LASSO regression effectively addresses multicollinearity and enhances model generalization. On the other hand, XGBoost automatically captures complex non-linear relationships and interaction effects. Finally, variables are selected through intersection screening and incorporated into the multivariate model, thereby improving clinical robustness and interpretability (28). Although esophageal dysfunction and smoking history showed significant differences in univariate analysis, they were excluded from the final model after LASSO and XGBoost calculations. This suggests their limited independent predictive value for the study outcome. After evaluating direct indicators like cough duration, the influence of esophageal dysfunction and smoking history further diminished, possibly exerting indirect effects through these mediating variables, ultimately resulting in low independent predictive contributions. Based on the above, a nomogram was constructed to visualize the complex multivariate regression equation. The ROC-AUC of 0.967 (95% CI 0.936–0.998) demonstrated excellent discriminatory ability. Calibration curves indicated good agreement between predicted and actual probabilities. The Hosmer-Lemeshow test yielded χ^2^ = 1.535, *P* = 0.992 > 0.05, confirming the model’s good accuracy and clear clinical significance. Nevertheless, this study has limitations: the small sample size and single-center design resulted in restricted representativeness, high homogeneity, and room for improvement in statistical power. Second, the study conducted internal validation of predictive models solely through ROC curves and calibration curves, lacking corresponding external validation. Furthermore, when LASSO and XGBoost yielded inconsistent variable selection results, the simple “overlap coverage” strategy, while enhancing robustness, may have discarded variables containing unique predictive information or critical interaction effects. Forcibly excluding such variables could lead to information loss, thereby reducing the model’s predictive accuracy and interpretive breadth. Future studies should refine the current design to enhance the scientific rigor of predictive models. To facilitate the translation of the current findings into routine clinical care, we strongly recommend that clinicians consult “*How to Apply This Knowledge to Clinical Practice*,” which offers systematic guidance on integrating research evidence into personalized patient management and clinical decision-making.

In conclusion, esophageal motility disorders, prolonged preoperative cough duration, higher Demetris score, lower levels of salivary SIgA, increased concentrations of gastric protease in saliva or sputum, and increased FeNO were all identified as independent predictors of persistent cough in patients with GERD and chronic cough after undergoing endoscopic minimally invasive treatment. The nomogram constructed based on these influencing factors has good predictive efficacy. The calibration curve and decision curve from internal validation both show that the logistic regression model has good calibration and clinical effectiveness. The results of this study provide support for the potential application value of this model in individualized prognosis assessment. However, which may have selection bias and the representativeness of the study population is limited. Second, only internal validation was performed, lacking external multi-center validation to verify model generalizability. Third, the hybrid machine learning feature selection may overlook potential interaction effects of some variables. Fourth, objective reflux measurements were used but long-term lifestyle modification data were not collected, which may affect outcome interpretation. Future studies should expand sample size, conduct prospective studies, and carry out multi-center external validation to improve model robustness and clinical applicability.

## Data Availability

The original contributions presented in this study are included in this article/supplementary material, further inquiries can be directed to the corresponding author.
